# Risky Sexual Behavior across Extremes of Wealth in sub-Saharan Africa: A Meta-Analysis of Demographic and Health Surveys

**DOI:** 10.4314/ejhs.v31i1.18

**Published:** 2021-01

**Authors:** Rahma Ali, Afework Tadele

**Affiliations:** 1 Department of Population and Family Health, Faculty of Public Health, Institute of Health, Jimma University

**Keywords:** Risky sexual behavior, Multiple sexual partner, Demographic and health survey, Sub-Saharan Africa, condom non-use

## Abstract

**Background:**

Risky sexual behavior increases the risk of contracting sexually transmitted disease including HIV and other reproductive health problems. There have been varying assumptions and different reported result explaining the relationship between risky sexual behavior and wealth. This review was intended to examine the disparity of risky sexual behavior among the two extremes of wealth in sub-Saharan African countries.

**Method:**

This study reviewed demographic and health survey reports of sub-Saharan African countries. We excluded older reports and reports published in languages other than English. Finally, reports from 16 countries were considered for review. Data were entered in excel and transported to stata for analysis. Metaprop and Metan command were used to compute proportions and odds ratio. Standard chi-square and I square tests were used to assess heterogeneity.

**Result:**

Pooled prevalence of having multiple sexual partner ranges from 2 to 12%. Over 80% of the countries reported that more than half of the individuals did not use condom at their last risky sexual intercourse. Poorest females were 0.62 [OR: 0.62, 95% CI (0.50, 0.78)] times less likely to have multiple sexual partners than males. Both males and females from the poorest wealth quantile had higher odds of not using condom at their last risky sexual intercourse, 1.41 [OR: 1.41, 95% CI (1.29, 1.53)], 1.41 [OR: 1.46, 95% CI (1.23, 1.73)], respectively.

**Conclusion:**

Multiple sexual partners is relatively low in the region. Condom non-use is high in both genders. Additionally, poorest males and females were at higher risk of not using a condom at last risky sexual intercourse.

## Introduction

Risky sexual behavior (RSB) is defined in several ways. The common definition is that it is a behavior that raises the susceptibility of an individual to sexual and reproductive health problems. This encompasses having sex at an early age, involving with multiple sexual partners (MSP), having sex while under the influence of drug and alcohol and unprotected sexual behaviors. RSB increases the probability of contracting a sexually transmitted infection (STI). Even nonfatal STI's are associated with adverse consequences like ectopic pregnancies and infertility. Human papilloma virus, which is the cause of genital warts, will also lead to cervical cancer. Likewise, the mere presence of an STI increases the likelihood of HIV transmission (1).

Globally, in 2018, 37.9 million of adults aged from 15 to 49 years were living with HIV. In African, nearly 1 in every 25 adults live with HIV. This contributes to over two-thirds of the world people living with HIV and sub-Saharan Africa takes the large share (2). Over 45% of the world's HIV infections and 53% of people living with HIV are found in Eastern and Southern Africa (3).

Even if the findings are not consistent, different studies hve been done so far on the link between wealth status and risky sexual behavior and infections like HIV. Poor countries have high HIV susceptibility and infection rates because of the limited resources the countries have to invest from prevention to curative services. At an individual level, risky behavior is mainly seen among the poor due to illiteracy and failure to negotiate for safer sex (4–7). On the contrary, risky sexual behavior like multiple and concurrent sexual partnerships is mainly seen among the wealthier population, especially men (8–11). Opposingly, a household with durables ownership is largely associated with reduced risky sexual behavior for both females and males (4).

As mentioned above, there have been contradicting assumptions and varying results about the relationship between wealth and risky sexual behavior. Hence, a single data set should not be used in generating public policies (12). The present review was intended to investigate the association between the two extremes of wealth index and RSB.

## Methods

**Data sources and study design**: DHS is a nationwide survey and the samples are representative from national to residence levels. It usually uses a stratified two-stage cluster sampling design. First, enumeration Areas (EA) are generally drawn from census files. Second in each selected EA, a sample of households is taken from an updated list of households. The programs use standard questionnaire which makes the collected data comparable across countries.

DHS surveys are designed to collect data on marriage, fertility, mortality, family planning, reproductive health, child health, nutrition, and HIV/AIDS. The data are collected through Household Questionnaire, Woman's Questionnaire, Man's Questionnaire, Biomarker Questionnaire, and Health Facility Questionnaire.

**Population**: Both men and women with an age range of 15- 49 were considered for analysis.

**Measurement**: Wealth index information was taken directly as it appears in the reports. DHS computs wealth index based on the number and kinds of consumer goods households own and housing characteristics. These scores are derived using principal component analysis (PCA). Wealth quintiles are compiled by assigning the household score to all household member. Then rank will be given to each person in the household population by their score. Finally, the distribution will be divided into five equal categories. Quantiles are expressed as poorest, poor, middle, rich and richest.

**The following operational definitions are used in this study.**

Risky sexual behavior (RSB): Defined as having multiple sexual partners in the past one year and last non-use of condom among individuals with MSP or who had higher risk intercourse in the past 12 months. The definition extends to, individuals who had intercourse in the past 12 months with a person who was neither their wife/husband nor lived with them.

**Inclusion and exclusion criteria**: This study reviewed demographic and health survey reports of sub-Saharan African countries published with in the past ten years and available at DHS web page(13). We excluded older dataset and countries survey reports written in languages other than English.

**Statistical analysis**: Data was entered into excel and transported to STATA™ version 14 for analysis. Proportions were computed using metaprop command. Metan command was used to compute odds ratio (OR) of having multiple sexual partners in the poorest and richest wealth quantiles. Random effect model was used and heterogeneity was assessed statistically using the standard chi-square and I square tests.

**Ethics approval**: the study uses publicly available DHS reports. Thus, ethical clearance procedure is held by institutions that commissioned, funded, or managed the surveys and ethics regulatory boards of each country.

## Results

**Included studies**: From the total 44 sub-Saharan Africa countries, 16 countries were included in the review. The rest 28 country reports were excluded because they were either too old datasets or the published reports did not have English version or they did not report the needed outcome variable. Namibia and Gambia did not have reports on last condom utilization among females with multiple sexual partners. A total of 334, 631 participants were included. Males were 109,948(32.9%) and 224,683(67.1%) were females.

**Prevalence of risky sexual behavior**: Throughout the region, pooled prevalence of having MSP ranges from 2% in Gambia and Ethiopia to 12% in Lesotho. For both male and female, women's having multiple sexual partner is as high as 7% in Lesotho and Liberia. Among men of age 15–49, Sierra Leone, Lesotho and Swaziland are the three leading countries with overall prevalence of 27%, 25% and 23%, respectively (Table 1).

**Table 1:** Proportion of risky sexual behavior among men and female age from 15–49 in Sub-Saharan African countries, 2010–2016

Country	Proportion of RSB		Multiple sexual partner (MSP) among				Non- use of condom among			
	
	MSP	non-use of condom	Male		Female		Male		Female	
			Poorest	richest	poorest	richest	poorest	richest	poorest	richest
**Ethiopia (2016)**	0.02	0.8	0.05	0.04	0	0	0.92	0.5	0.97	0.69
**Gambia (2013)**	0.02	0.81	0.09	0.08	0	0	0.9	0.63	-	-
**Ghana (2014)**	0.05	0.82	0.11	0.15	0.01	0.01	0.93	0.67	*	0.41
**Kenya (2014)**	0.07	0.56	0.12	0.14	0.01	0.02	0.69	0.53	0.82	0.52
**Lesotho (2014)**	0.12	0.39	0.21	0.33	0.06	0.08	0.49	0.29	0.74	0.34
**Liberia (2013)**	0.1	0.78	0.21	0.15	0.03	0.09	0.83	0.65	0.96	0.64
**Malawi (2015/16)**	0.04	0.71	0.12	0.12	0.01	0.01	0.75	0.59	0.77	0.67
**Namibia (2013)**	0.05	0.28	0.09	0.11	0.01	0.03	0.32	0.22	-	-
**Nigeria (2013)**	0.05	0.79	0.16	0.12	0.01	0.02	0.99	0.56	0.91	0.47
**Rwanda (2014/15)**	0.02	0.64	0.05	0.07	0.01	0.01	0.85	0.45	0.64	0.34
**Sierra Leone(2013)**	0.11	0.9	0.22	0.3	0.04	0.08	0.93	0.77	0.95	0.94
**South Africa (2016)**	0.08	0.38	0.13	0.13	0.04	0.02	0.35	0.35	0.52	0.39
**Swaziland (2006/07**	0.11	0.45	0.23	0.22	0.02	0.02	0.57	0.38	0.64	0.32
**Uganda (2011)**	0.06	0.67	0.18	0.22	0.02	0.02	0.85	0.69	0.83	0.72
**Zambia (2013/14**	0.08	0.71	0.17	0.11	0.01	0.02	0.75	0.58	0.87	0.7
**Zimbabwe(2010/11)**	0.05	0.65	0.12	0.11	0.01	0.01	0.81	0.53	*	*
**pooled prevalence**	0.04	0.72	0.11	0.1	0.01	0.01	0.94	0.57	0.88	0.7

- Gambia and Namibia did not report condom utilization among females

* DHS report suppress it because the category had less than 25 unweighted cases

Around 80% of the countries reported more than half of their people did not use condom at their last risky sexual intercourse. Overall, 72% for men and 77% of females did not use condom at their last risky sexual intercourse. Sierra Leone is the first country with 95% of its females and 87% of its males reported they did not use condom at their last risky sexual intercourse (Table 1). The poorest males had a higher proportion of non-use of condom, which is 94% followed by 88% among the poorest females (Table 1).

**Figure 2:**
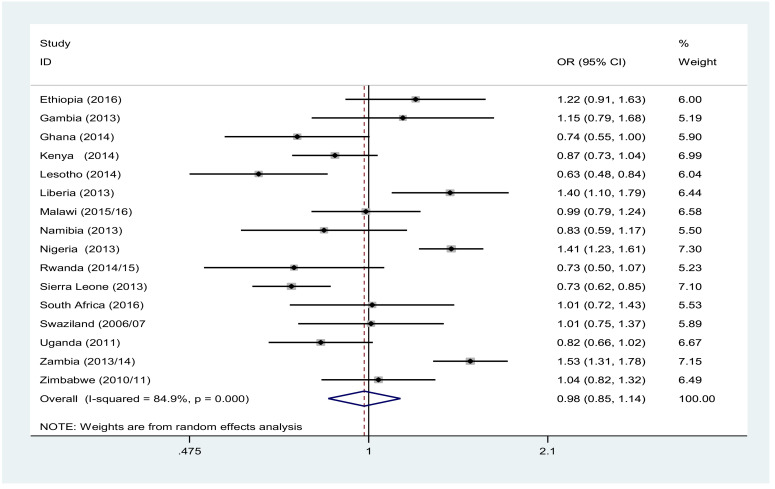
Forest plot of multiple sexual partner likelihood among males 15–49 years of age living in the poorest versus those living in the richest households in Sub-Saharan Africa, A meta-analysis, 2010–2016.

**Figure 3:**
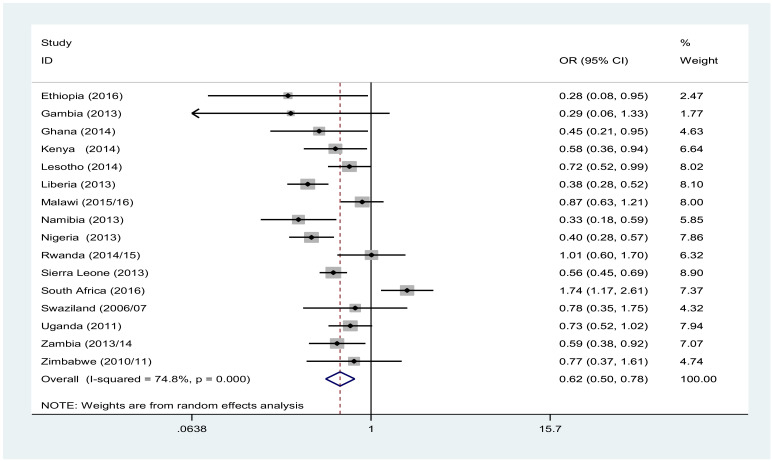
Forest plot of multiple sexual partner likelihood among females 15–49 years of age living in the poorest versus those living in the richest households in Sub-Saharan Africa, A meta-analysis, 2010–2016.

Overall, wealth did not show significant association with MSP among males. However, we observed inconsistent results across countries. In Lesotho and Sierra Leone, the males from the poorest wealth quantile were less likely to have MSP than the richest. In contrast, poorest males living in Liberia, Nigeria and Zambia were more likely to have MSP than the richest (Figure 2). Females from poorest wealth were less likely to have MSP, and this was evident in most of the countries. The pooled result reveal that females in the poorest wealth quantile were 0.62 [OR: 0.62, 95% CI (0.50, 0.78)] times less likely to have MSP than the richest one (Figure 3).

**Figure 4:**
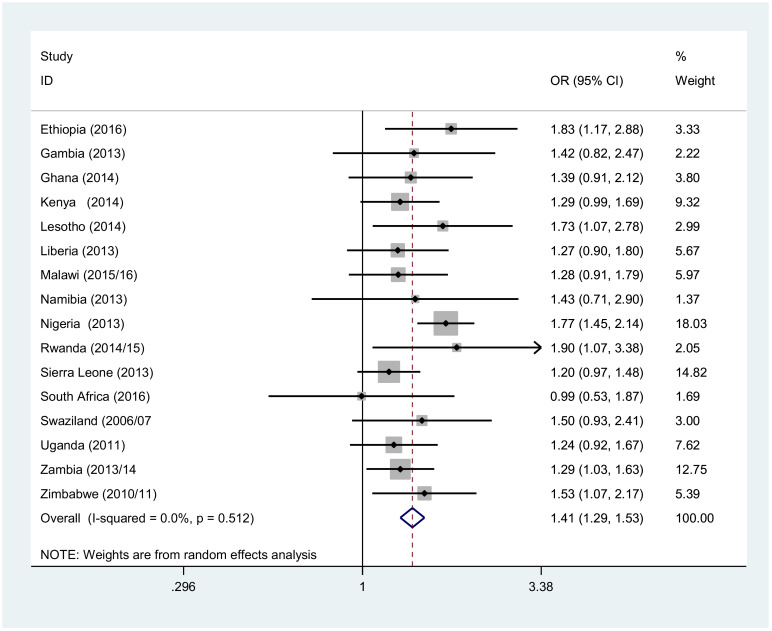
Forest plot of condom not use likelihood among males 15–49 years of age living in the poorest versus those living in the richest households in sub Saharan Africa, A meta-analysis, 2010–2016.

**Figure 5:**
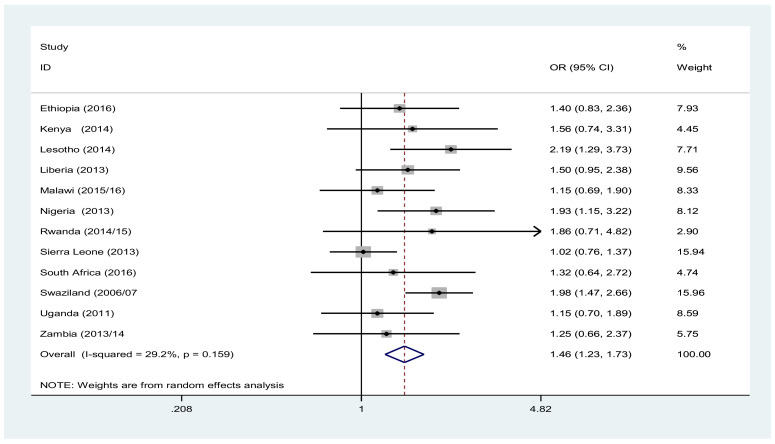
Forest plot of condom not use likelihood among females 15–49 years of age living in the poorest versus those living in the richest households in sub Saharan Africa, A meta-analysis, 2010–2016.

Throughout the region, except in South Africa, males from the poorest wealth quantile were more likely to not use condom at their last risky sexual intercourse. In Ethiopia and Rwanda, poorest males had 1.83 [OR: 1.83, 95% CI (1.17, 2.88)] and 1.9 [OR: 1.9, 95% CI (1.07, 3.38)] times higher odds of not using condom than the males in richest wealth quantile (Figure 4). Likewise, in all countries, the females in the poorest wealth quantile had higher odds of not using condom at their last risky sexual intercourse. Overall, the poorest females had 1.46 [OR: 1.46, 95% CI (1.23, 1.73)] times higher odds of not using condom at their last risky sexual act than their counterpart (Figure 5).

## Discussion

Preventing HIV in SSA has a far-fetched impact on the overall global burden. Different prevention strategies have been implemented so far in the region (14). Yet, in this review, 72% of individuals with risky sexual intercourse did not use condom. This result is much higher than even the DHS review report on adolescents condom use from 2003 to 2009 (15). This might be the reason HIV infection is increasing in some countries of the region (16) and even doubling among adults in Ethiopia (17). Specifically, around 77% of females in the region did not use condom at their last risky sexual intercourse. This result is higher than the males. This explains the power inequalities and subordinate position of women in SSA, which makes them less influential in negotiating safe sex, including condom usage (18). In another way, the pooled MSP prevalence is 4% which is relatively low from past pooled reports in the region (19,20).

The pooled result of being very poor or very wealthy does not have a significant association with MSP among males living in sub-Saharan African countries. Even if the direction of association varies, few countries showed significant association. Males from the poorest wealth quantile living in Lesotho and Sierra Leone were less likely to have MSP and the poorest males in Liberia, Nigeria and Zambia were more likely to have MSP. Contradicting results were reported on many studies assessing the relationship between wealth and MSP. A similar DHS review done in four Africa countries also shows that wealth does not have a significant effect on male adolescents' risky sexual behavior including condom utilization (21). A result from Tanzania's study shows that wealth increment reduces the rate of having multiple sexual partners in males (22). Opposingly, wealthy men in Cameroon were more likely to have multiple sexual partners (11).

Both males and females from the poorest wealth quantile were more likely not use a condom at their last risky sexual intercourse. Similarly, in a study from Tanzania, non-use of condoms decreases with an increase in wealth for both genders (22). Also, in Malawi and Uganda, poorest adolescents had the lowest odds of using condoms (21). Due to language limitation of the authors, the study excluded reports written in languages other than English. This might affect the pooled results of the study.

In this review, MSP is relatively low in the region for both males and females. However, incredibly very high percent of male and female did not use condom at their last risky sexual intercourse. Also, poorest males and females were at higher risk of not using a condom. Beside what has been done so far, special focus should be given on minimizing risky sexual behavior specially condom utilization. Additionally, special focus should be given for poorest individuals in the region while designing programs and different interventions.

## Figures and Tables

**Figure 1:**
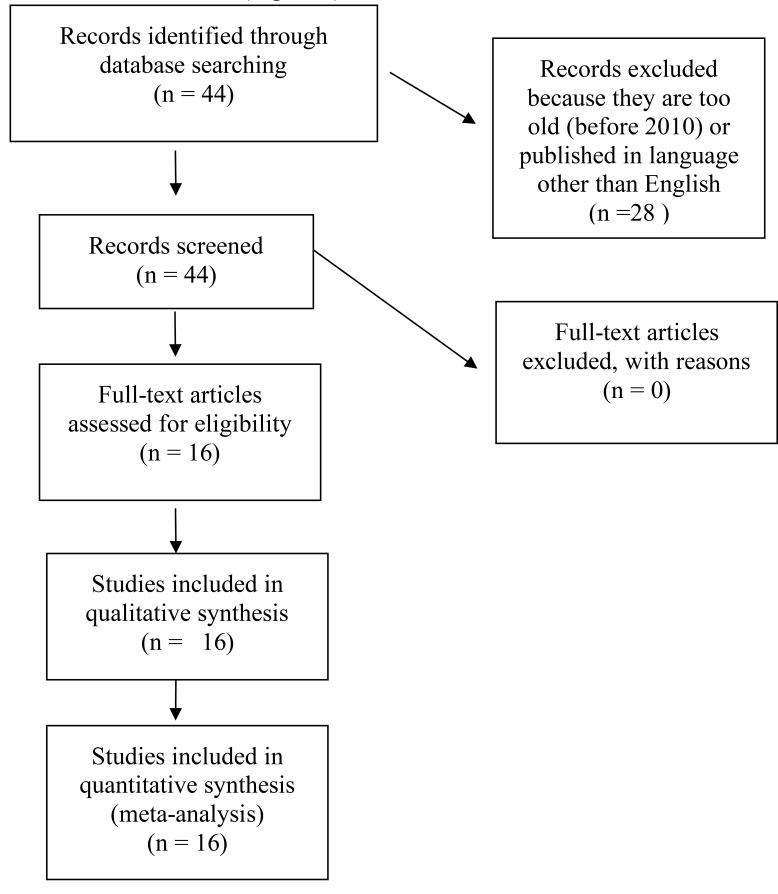
Flowchart of literature review process risky sexual behavior and wealth in Sub-Saharan Africa

## References

[1] (2019). Risky Sexual Behaviour in Adolescence.

[2] WHO | HIV/AIDS (2019). WHO.

[3] (2018). UNAIDS data.

[4] Lucas AM, Wilson NL (2018). Schooling, wealth, risky sexual behavior, and hiv/aids in sub-saharan africa.

[5] Butler C (2000). HIV and AIDS, poverty, and causation. Lancet.

[6] Fenton L (2004). Preventing HIV/AIDS through poverty reduction: the only sustainable solution?. Lancet.

[7] Pascoe SJS, Langhaug LF, Mavhu W, Hargreaves J, Jaffar S, Hayes R (2015). Poverty, Food Insufficiency and HIV Infection and Sexual Behaviour among Young Rural Zimbabwean Women.

[8] O'Farrell N (2001). Poverty and HIV in sub-Saharan Africa. Lancet.

[9] Shelton JD, Cassell MM, Adetunji J (2005). Is poverty or wealth at the root of HIV?. Lancet.

[10] Kongnyuy EJ, Wiysonge CS, Mbu RE, Nana P, Kouam L (2006). Wealth and sexual behaviour among men in Cameroon. BMC International Health.

[11] Oliver S, Dickson K, Bangpan M (2015). Systematic reviews : making them policy relevant A briefing for policy makers and systematic reviewers.

[12] The DHS Program - Demographic and Health Survey (DHS).

[13] Kharsany ABM, Karim QA (2016). HIV Infection and AIDS in Sub-Saharan Africa: Current Status, Challenges and Opportunities. Open AIDS J.

[14] Berhan Y, Berhan A (2015). A Meta-Analysis of Risky Sexual Behaviour among Male Youth in Developing Countries. AIDS Res Treat.

[15] (2017). Progress towards the 90-90-90 targets Ending AIDS GLOBAL AIDS UPDATE | 2017 [Internet].

[16] Girum T, Wasie A, Worku A (2018). Trend of HIV/AIDS for the last 26 years and predicting achievement of the 90-90-90 HIV prevention targets by 2020 in Ethiopia: a time series analysis. BMC Infect Dis.

[17] Tsai AC, Subramanian SV (2012). Proximate context of gender-unequal norms and women's HIV risk in sub-Saharan Africa. AIDS.

[18] E Mhele K (2017). Covariates of Multiple Sexual Partnerships among Sexually Active Men in Lesotho. Afr J Reprod Health.

[19] Madise N, Zulu E, Ciera J, Health AJR (2007). Is Poverty a Driver for Risky Sexual Behaviour?. Evidence from National Surveys.

[20] Silas J (2013). Poverty and Risky Sexual Behaviours.

